# Supplementary material for: Learning curve and surgical outcome of robotic assisted colorectal surgery with ERAS program
Source: Sci Rep. 2022 Nov 29;12:20566. doi: 10.1038/s41598-022-24665-w (PMC9709162; doi:10.1038/s41598-022-24665-w)
Supplement: Supplementary file 2 — Supplementary Information 2. [file 41598_2022_24665_MOESM2_ESM.zip › RAL Raw data-Ñ[▒K/074/HS_ñw╝╨░O▒Kñσ.pdf]

正式摘要(電子簽章：000000214874900) W52 / W52 ☐ 英文標題(注意:無英文姓名-請補病人基本檔!)	☐【讀取】憑證	

臺中榮民總醫院

出院病歷摘要

轉入號： 轉出號：

[redacted]

**(8)**流水編號	
**(9)**入院日期	110/04/16 (2021) 直腸外科 W52 - 143
**(10)**轉科日期	
**(11)**出院日期	110/04/20 (2021) 住院天數計 4 日

診斷

- 入院 :1. Rectal tumor, adenocarcinoma, 10cm FAV, with partial obstruction
- 出院 :1. Rectal tumor, adenocarcinoma, 10cm FAV, with partial obstruction
  - s/p Robot Assisted Colectomy(L) on 2021/04/17

主訴

- Bloody stool for half year

病史

- .This 68 year-old female had history of hemorroid
  She had bloody stool for half year, and went to LMD. Coloscope was arranged, and annular tumor with partial obstruction was found. Then she was refered to our CRS OPD. Under impression of rectum tumor with partial obstruction, she was admitted to our ward for further evaluation and treatment.

體檢發現

- ..ARRANGE CT
  - General appearance :
    - easy looking , conscious clear , without cardiopulmonary distress
    - Vital signs自動帶入date 體溫(BT) 脈搏(P) 呼吸(R) 血壓(BP) 身高(BH) 體重(BW) BMI
      20210416 36.5℃ 88次/分 18次/分 138/79mmHg 158.4cm 46.9kg 18.7
    - Vital signs: B.P. - mmHg , P.R. 5 /min , R.R. 5 /min
  - HEENT :
    - Eyes: no anemic conjunctiva, no icteric sclera, normal EOM
    - ENT: no otorrhea, no rhinorrhea, no erythematous mucosa, no injected tonsil, no deformity
  - Neck: supple, freely movable, trachea midline,no deformity, no engorged jugular vein , no palpable thyroid or lymph node enlargement
  - Chest:
    - Inspection: Grossly normal, symmetric and fully expansion, no accessory muscle used, no operation scar, no spider angiomata, no gynecomastia
    - Palpation: no palpable mass or nodule, no subcutaneous emphysema, no tenderness
    - Ausculation: clear breathing sound
  - Heart:
    - Percussion: normal heart size
    - Ausculation: regular heart beat, normal S1, S2, no gallop, no murmur
  - Abdomen:
    - Inspection: in
      - flat contour, symetrically, no scar, no stria, no dilated veins, no rashes
    - Ausculation: normoactive bowel sound, no abdominal bruits
    - palpation: soft, no muscle guarding or rigidity, no local tenderness, no rebounding pain, no palpable mass, no palpable pulsation
    - Percussion: no shifting dulllness, no hepatomegaly, no splenomagaly
  -

Peripheral pulsation :	CA	BA	RA	FA	PA	PTA	DPA
Right side :	++	++	++	++	++	++	++/+++
Left side :	++	++	++	++	++	++	++/+++

  - Genito-anus: no tenderness or palpable mass lesion or rectal shelf, normal external genitalia, normal anal tone, brown stool

- **CRS CONDITION**
    -
        - Lung

手術日期及方法

- 手術日期及方法20210417 Robot Assisted Colectomy(L)
- 手術結果及發現：
    - 1. One 4x4 cm tumor located at middle rectum colon 2. There was no palpable liver metastasis, no visible peritoneal seeding. 3. High ligation (+) above the left colic artery.

住院治療經過

- After admission, there was no contraindication for operation and Robot Assisted Colectomy(L) was performed smoothly on 04/17. She tolerated the procedure and no post-op complication was noted. She recovered well and oral intake was smooth since 04/18. Under the stable condition, she was discharged and would be followed-up at OPD.

合併症

- nil

一般檢查

- 累積報告：

DATE	TIME	ALB	TP	BIL.T	BIL.D	ALKP	AST	ALT	LDH	CHOL	TG	HDL-C	LDL	UA	NH3	AMY
1100417	1425	3.3		0.49	0.12	65	14	10								
1100420	0850	3.1		0.5			10	5								

DATE	TIME	WBC	RBC	HGB	HCT	MCV	PLT	BLST	NEUT	BAND	SEG	LYM	MON	EOS	BAS
1100417	1425	15300		12.7			256		94.2			4.2	1.4	0.1	0.1
1100420	0908	14780	3.88	11.9			215		91.5			5.7	2.5	0.2	0.1

DATE	TIME	NA	K	CL	CA	BUN	CREAT	Mg	P	CRP	ESR	PCT	LIPASE	LACTATE
1100417	1425	137	3.0			6	0.70	1.8	2.0					
1100420	0850	132	3.1	93	8.1	7	0.68							

- 放射線報告 :檢驗項目 **:CT ABDOMEN-SURVEY** 報告日期 **:1100419**

主　訴：
Result:Y
Finding:
Imaging Report Form for Colorectal Carcinoma (AJCC 8th)
1. Imaging Date: 20210416
Imaging Modality, Imaging by
CT scan
Imaging Protocol: CT scan of abdomen with IV and oral contrast enhancement:
2. Tumor location / Size
Location:
Rectum
Size:
Measurable: 4.4 cm (largest diameter)
3. Tumor invasion
Tumor within colonic or rectal wall
4. Regional nodal metastasis
No
5. Distant metastasis (In this study)
No or Equivocal
6. Other findings: > A case of rectal cancer for pre-OP survey
\> Circumscribed wall thickening over proximal rectum about 4.4cm(axi:61-66) is compatible with rectal cancer
\> Atherosclerotic changes of aorta
\> The liver, gallbladder, pancreas, spleen, both kidneys are without specific abnormality
\> No significant lymphadenopathy in the abdomen or retroperitoneum is identified
\> DJD change of thoracic and lumbar spine with osteophytes formation.
\> A 0.9cm nodular lesion in RLL, suggest F/U
Impression:
Rectal cancer, T1/T2N0M0

特殊檢查

病理報告

- Pending

其它

出院時情況

- 3.改門診治療

出院指示

-

藥名	劑量	劑量單位	用法	頻次	數量	數量單位	現況	天數	單位
Ultracet tab	1	TAB	PO	Q6H	28	TAB	TH	7	天
Mag. Oxide tab	2	TAB	PO	TID	42	TAB	TH	7	天
(□)Takepron OD tab 30mg	1	TAB	PO	QDAC	7	TAB	TH	7	天

出院計劃

主治醫師 2062F 蔣鋒帆 / 住院醫師 2334J 陳勇先

正式摘要(電子簽章：000000214874900)

W52 / W52

doctorRank:B
